# When Neonates Require Anesthesia for the Management of Airway-Related Problems

**DOI:** 10.7759/cureus.98631

**Published:** 2025-12-07

**Authors:** Beatriz Maio, Marisa Silva

**Affiliations:** 1 Anesthesiology, Hospital da Luz Lisboa, Lisbon, PRT; 2 Anesthesiology, Unidade Local de Saúde de São José, Hospital Dona Estefânia, Lisbon, PRT

**Keywords:** airway management, airway topical anesthesia, anesthetic plan, dexmedetomidine, ketamine, neonatal anesthesia

## Abstract

Suspension laryngoscopy is a valuable tool for the diagnosis and treatment of airway pathologies such as subglottic stenosis in neonates. Propofol and remifentanil sedation are commonly used drugs for this purpose, but there is still concern about their dose-dependent respiratory depression and safety profile in neonates. This clinical case-based discussion presents a premature neonate with respiratory failure and stridor, scheduled for a suspension laryngoscopy. An anesthetic plan using sevoflurane, ketamine, and dexmedetomidine was chosen. This combination preserves airway tone and minimizes the risk of respiratory depression and desaturation, making it an ideal choice for neonates with airway abnormalities.

## Introduction

Subglottic stenosis in neonates is a serious condition that can lead to significant airway compromise and respiratory distress. While it can be congenital, acquired forms of subglottic stenosis are more common, typically due to prolonged intubation or trauma to the airway [1]. This article discusses a clinical scenario involving a two-month-old (22-day-old corrected gestational age) premature neonatal patient with congenital subglottic membrane and acquired subglottic stenosis secondary to mechanical ventilation. The patient was scheduled for a suspension laryngoscopy to assess and potentially treat the underlying stenosis. The primary concern was the unfavorable respiratory physiology typical of premature infants, including structurally immature lungs and immature control of breathing. In addition, the risk that endotracheal intubation might fail to pass beyond the obstruction was an important consideration. Therefore, an anesthetic plan combining sevoflurane, ketamine, and dexmedetomidine was selected to minimize the risk of compromising spontaneous ventilation. This drug combination, together with topical airway anesthesia, helps preserve airway tone and reduces the risk of respiratory depression. It also provides effective analgesia, with each medication offsetting the other’s side effects, making it a safer alternative to many commonly used agents in neonatal airway management.

## Case presentation

This article presents a clinical case involving a preterm neonate born at 33 weeks’ gestation from a monochorionic, monoamniotic twin pregnancy, with a low birth weight of 1740 g. Due to neonatal sepsis, he was intubated and remained on invasive ventilation for 15 days. He was discharged home on day 55 after birth with 2465 g. Eight days after discharge, he presented in the emergency department with respiratory failure and biphasic stridor, suggesting airway obstruction. Emergent suspension laryngoscopy was proposed to diagnose and potentially treat the underlying etiology.

Given the anticipated difficult airway, specifically the risk of not being able to advance an endotracheal tube past the obstruction and the potential for rapid desaturation in a premature neonate with immature ventilatory control, and the requirement to assess the biphasic stridor under spontaneous breathing, the chosen anesthetic approach combined ketamine, dexmedetomidine, and topical airway anesthesia with atomized lidocaine. Sedation was further supported with short periods of intermittent sevoflurane administration.

The desired level of anesthetic depth was achieved by carefully titrating ketamine and dexmedetomidine administration, which enabled the procedure to be performed safely while carefully monitoring the patient's respiratory status (pulse oximetry and end-tidal CO₂). A low-dose intramuscular bolus injection of ketamine and dexmedetomidine 2/2 (ketamine 2 mg/kg and dexmedetomidine 2 µg/kg) was initially administered according to local practice and clinical expertise, complemented by low-dose sevoflurane administration through a facial mask. A peripheral IV catheter was secured, and the patient was adequately sedated while maintaining spontaneous ventilation. Subsequently, a nasal cannula was used instead of a face mask to administer oxygen. In order to determine the patient's tolerance to the upcoming stimuli, a videolaryngoscopy was performed beforehand. The glottic opening was visualized, but the subglottic stenosis was critical. Atomized lidocaine was administered to anesthetize the upper airway and periglottic structures.

**Figure 1:**
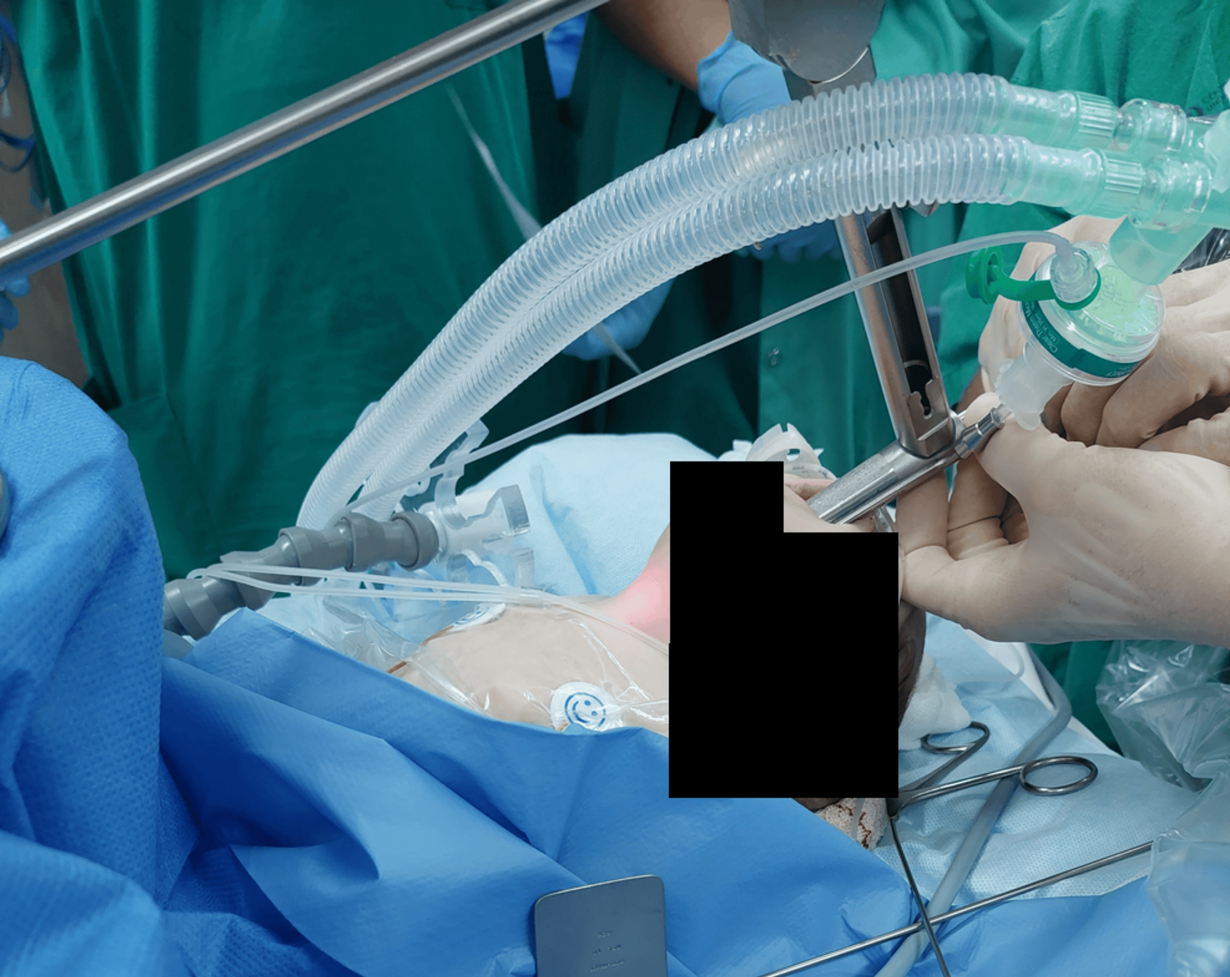
Suspension laryngoscopy in the spontaneously breathing neonate Suspension laryngoscopy in the spontaneously breathing neonate using topical airway anesthesia, intermittent low-dose sevoflurane, and a combination of ketamine and dexmedetomidine titrated to achieve the required depth of anesthesia.

Suspension laryngoscopy was then performed, during which two balloon subglottic dilations were performed, and additional small boluses of IV ketamine and dexmedetomidine were given and titrated up to a total of 1/1 (ketamine 1 mg/kg and dexmedetomidine 1 µg/kg). Sedation was supplemented by short periods of intermittent low-dose sevoflurane administration (Figure 1).

The patient maintained spontaneous ventilation throughout the procedure, with a minimum oxygen saturation of 88% recorded during the balloon dilation phase. Hemodynamic stability was preserved throughout the procedure. No adverse events were registered. At the end of the procedure, the neonate was transferred to the neonatal intensive care unit, breathing spontaneously with supplemental oxygen delivered via nasal cannula.

## Discussion

Suspension laryngoscopy is an important tool for diagnosing and treating airway pathologies like subglottic stenosis in neonates. However, it is a challenging procedure that requires precise anesthesia management, especially in neonates who have anatomically and physiologically difficult airways. Maintaining airway patency, spontaneous breathing, and adequate oxygenation throughout the procedure are the mainstay of anesthesia management [2].

Propofol and remifentanil are the most widely used drugs for pediatric procedural sedation due to their rapid onset and short duration of action [3]. Nevertheless, there is apprehension about their dose-dependent respiratory depression and safety profile in neonates, whereas other drugs may be safer for sedation. Additionally, propofol and remifentanil are associated with unpredictable pharmacodynamics in neonates, with significant interindividual variability in both sedative effect and adverse events, with concerns regarding hemodynamic stability, respiratory depression, and chest wall rigidity [4,5].

Ketamine, an N-methyl-D-aspartate receptor antagonist, is particularly useful in anesthesia for neonatal airway procedures. It provides effective sedation while maintaining airway reflexes and spontaneous ventilation, which is essential for neonates who are at risk of airway obstruction or collapse without appropriate airway support. Furthermore, ketamine acts as a bronchodilator, offering additional benefits for neonates with compromised airways. Finally, it provides analgesia, which is critical during painful procedures such as rigid bronchoscopy and laryngeal suspension [6].

Dexmedetomidine, an alpha-2 adrenergic agonist, provides sedation without significant effects on airway patency. The minimal impact on respiratory drive makes its use in pediatric airway procedures beneficial. Furthermore, dexmedetomidine’s sedative effects allow for smooth transitions between sedation and wakefulness, minimizing the need for additional airway interventions [7,8].

The combination of these two agents has synergistic effects and antagonizes each other’s adverse effects: dexmedetomidine reduces ketamine-induced tachycardia, emergence phenomena, and sialorrhea, while ketamine counteracts dexmedetomidine-induced bradycardia and hypotension [1].

Sevoflurane is a volatile anesthetic widely used in pediatric anesthesia due to its ability to rapidly induce and maintain general anesthesia while also allowing for rapid and precise control over anesthetic depth. The continuous and sole use of sevoflurane is challenging in these cases due to frequent airway-interface disconnections and the risk of dose-dependent respiratory depression, particularly in neonates [2]. However, the intermittent use of low doses of sevoflurane helps maintain an adequate anesthetic depth while benefiting from its important bronchodilator effects.

## Conclusions

The choice of ketamine and dexmedetomidine with intermittent low doses of sevoflurane combined with topical anesthesia of the airway can provide balanced sedation and pain management for airway procedures. This combination preserves airway tone and minimizes the risk of respiratory depression, making it an ideal choice for neonates with airway abnormalities. These two agents also have synergistic effects, and when given together, antagonize each other’s adverse effects. The success of ketamine and dexmedetomidine in this clinical scenario aligns with the growing body of evidence supporting its use in pediatric airway procedures, particularly those involving neonates with challenging airway anatomy and physiology. Future studies addressing their individual or combined use, specifically in neonates, would be beneficial.
